# Microbiological Contamination of the Office Environment in Dental and Medical Practice

**DOI:** 10.3390/antibiotics10111375

**Published:** 2021-11-10

**Authors:** Alexandre Baudet, Monique Guillaso, Léonie Grimmer, MEDIQAI Study Group, Marie Regad, Arnaud Florentin

**Affiliations:** 1 Faculté d’Odontologie, Université de Lorraine, F-54505 Vandœuvre-lès-Nancy, France; 2 Service d’Odontologie, CHRU-Nancy, F-54000 Nancy, France; 3 APEMAC, Université de Lorraine, F-54505 Vandœuvre-lès-Nancy, France; arnaud.florentin@univ-lorraine.fr; 4 Département d’Hygiène, des Risques Environnementaux et Associés aux Soins, Faculté de Médecine, Université de Lorraine, F-54505 Vandœuvre-lès-Nancy, France; guillaso_monique@yahoo.fr (M.G.); leonie.grimmer@univ-lorraine.fr (L.G.); m.regad@chru-nancy.fr (M.R.); 5 Département Territorial d’Hygiène et de Prévention du Risque Infectieux, CHRU-Nancy, F-54505 Vandœuvre-lès-Nancy, France

**Keywords:** environmental microbiology, environmental contamination, indoor air, dental offices, general practitioner offices, antibiotic resistance

## Abstract

The microbiological contamination of the environment in independent healthcare facilities such as dental and general practitioner offices was poorly studied. The aims of this study were to describe qualitatively and quantitatively the bacterial and fungal contamination in these healthcare facilities and to analyze the antibiotic resistance of bacterial pathogens identified. Microbiological samples were taken from the surfaces of waiting, consulting, and sterilization rooms and from the air of waiting room of ten dental and general practitioner offices. Six surface samples were collected in each sampled room using agar contact plates and swabs. Indoor air samples were collected in waiting rooms using a single-stage impactor. Bacteria and fungi were cultured, then counted and identified. Antibiograms were performed to test the antibiotic susceptibility of bacterial pathogens. On the surfaces, median concentrations of bacteria and fungi were 126 (range: 0–1280) and 26 (range: 0–188) CFU/100 cm^2^, respectively. In indoor air, those concentrations were 403 (range: 118–732) and 327 (range: 32–806) CFU/m^3^, respectively. The main micro-organisms identified were Gram-positive cocci and filamentous fungi, including six ubiquitous genera: *Micrococcus*, *Staphylococcus*, *Cladosporium*, *Penicillium*, *Aspergillus*, and *Alternaria*. Some antibiotic-resistant bacteria were identified in general practitioner offices (penicillin- and erythromycin-resistant *Staphylococcus aureus*), but none in dental offices. The dental and general practitioner offices present a poor microbiological contamination with rare pathogenic micro-organisms.

## 1. Introduction

Due to the nature of their activities, healthcare facilities are at higher risk of presenting microbiological contamination, which is in line with the infectious nature of patients and interventions [1]. Medical environments receiving ill patients can be contaminated by skin contact, liberation of skin squamæ [2], bio-aerosols, or droplets (talking, breathing, sneezing, or coughing) that contaminate indoor air and surfaces [1]. Dental offices present significant bio-aerosol contamination due to the widespread use of high-speed dental turbines, hand pieces, and mechanical scalers, which produce aerosols from supply water and mouth fluids mainly contaminated with bacteria [3]. In that respect, healthcare workers and patients are exposed to numerous infectious agents including drug-resistant bacteria [4]. Exposure to bacteria and fungi present on environmental surfaces—medical equipment and housekeeping surfaces—can conduct to cross-transmission of micro-organisms and healthcare-associated infections [5].

Although monitoring the environmental contamination of hospitals has become routine, the microbiological contamination of the environment in independent healthcare facilities is not subject to specific supervision. Nevertheless, healthcare environments may contribute to the spread of healthcare-associated infection among patients [2] and occupationally acquired infection among workers [6].

Bio-aerosol composition produced during dental treatments was extensively studied [1]. However, other microbiological contamination in independent healthcare facilities—both in dental and general practitioner (GP) offices—was poorly studied. To the authors knowledge, only four other study groups have sampled surfaces in very few dental offices. Pasquarella’s study sampled only two surfaces in consulting rooms of ten dental offices without studied the antibiotic resistance [7]. The three other studies sampled surfaces in dental consulting rooms but to surveyed only methicillin-resistant *Staphylococcus aureus* [4,8,9]. In a previous study, we sampled indoor air and only two surfaces in two dental and GP offices to identify the micro-organisms and the antibiotic resistance of bacterial pathogens but with other sampling methods (cyclonic liquid air sampler and swabs) [10].

The aim of this study was to give a qualitative and quantitative description of bacterial and fungal contamination in dental and GP offices in France. A second aim was to analyze the antibiotic resistance of bacterial pathogens identified in those healthcare facilities.

## 2. Results

### 2.1. Quantitative Analysis

**Figure 1:**
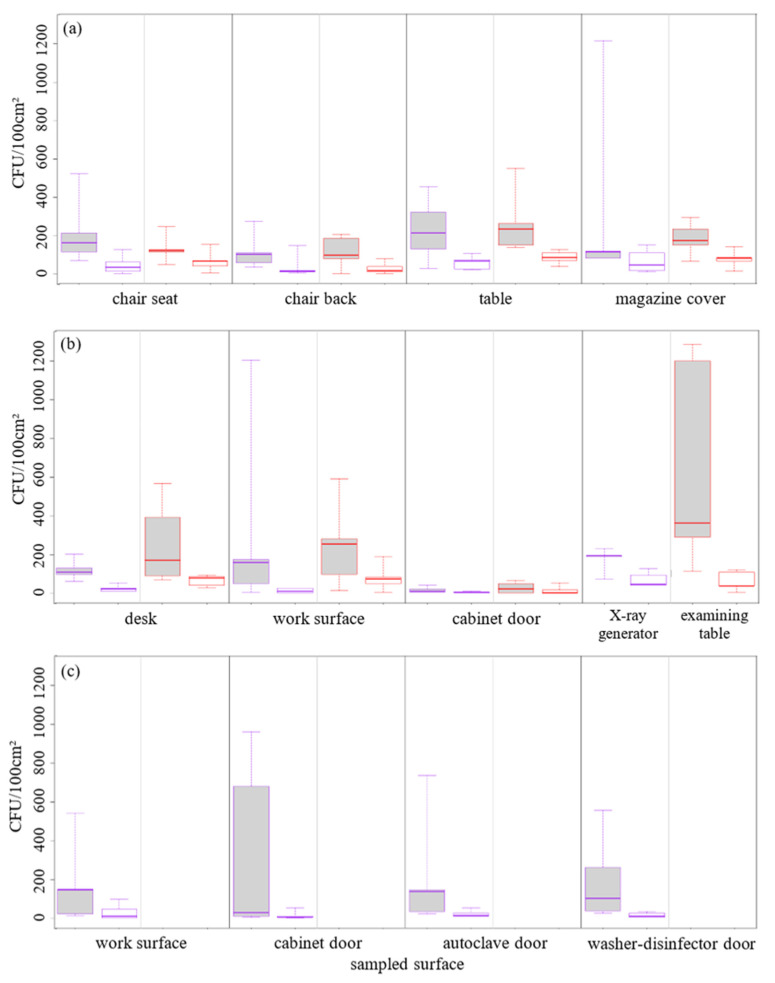
Bacterial (grey) and fungal (white) contamination in (**a**) waiting rooms, (**b**) consulting rooms, and (**c**) sterilization rooms of dental (purple borders) and general practitioner (red borders) offices.

Bacterial and fungal cultures from contact plates showed median concentrations of 126 (range: 0–1280) and 26 (range: 0–188) CFU/100 cm^2^, respectively. Those bacterial and fungal concentrations were distributed as follows: 132 (range: 0–1212) and 52 (range: 0–152) CFU/100 cm^2^ in waiting rooms; 110 (range: 0–1280) and 26 (range: 0–188) CFU/100 cm^2^ in consulting rooms; 118 (range: 4–960) and 8 (range: 0–96) CFU/100 cm^2^ in sterilization rooms. The details for each sampled surface were presented in Figure 1.

Bacterial and fungal cultures from sampled air in waiting rooms revealed median concentrations of 403 (range: 118–732) and 327 (range: 32–806) CFU/m^3^, respectively.

There was no statistical difference in microbiological contamination between the surface and air samples of dental and GP rooms (Supplementary Materials Table S1). The only significant difference was a higher fungal contamination in dental waiting rooms compared with sterilization rooms (*p* < 0.04) (Supplementary Materials Table S2). Offices with daily vs. non-daily deep wet cleaning had surfaces significantly less contaminated by fungi (18 vs. 64 CFU/100 cm^2^; *p* < 0.002) but similarly contaminated by bacteria (128 vs. 122 CFU/100 cm^2^; *p* = 0.7).

### 2.2. Qualitative Analysis

The most prevalent micro-organisms in dental and GP samples were Gram-positive cocci (from 59 to 67% of identified microbiota), mainly *Micrococcus*, *Staphylococcus*, and *Kocuria*, followed by filamentous fungi (from 25 to 30%), mainly *Cladosporium* and *Penicillium*; Gram-negative bacilli (from 2 to 14%), mainly *Stenotrophomonas* and *Pseudomonas*; and endospore-forming Gram-positive bacilli (from 1%) with *Bacillus*.

Table 1 presents micro-organisms identified from the air and surfaces of offices sampled: 27 bacterial species from 14 genera, and 18 filamentous fungal species from 14 genera. *Micrococcus*, *Staphylococcus*, *Cladosporium*, *Penicillium*, *Aspergillus*, and *Alternaria* were found in all healthcare offices. The highest microbial diversities were found in the GP consulting rooms for bacteria (16 bacterial species from eight genera) and in the dental waiting rooms for fungi and yeasts (14 fungal species from 11 genera).

### 2.3. Antibiotic-Resistant Bacteria

Concerning antibiotic resistance, the bacterial pathogens were tested including mainly several strains of *Staphylococcus* (*S. aureus*, *S. haemolyticus*, *S. sciuri*) and *Stenotrophomonas maltophilia*. Penicillin-resistant *S. aureus* were found in the air of a GP waiting room. In another GP office, *S. aureus* resistant to penicillin and erythromycin, and *S. haemolyticus* resistant to methicillin and erythromycin were found respectively on the table and in the air of the waiting room. No antibiotic-resistant bacteria were found in dental offices sampled.

## 3. Discussion

This environmental study shows a poor microbiological contamination in dental and GP offices, with few pathogenic micro-organisms and rare antibiotic-resistant bacteria. On the surfaces of consulting rooms, the median bacterial contamination was 110 CFU/100 cm^2^. Higher median contamination was found on the surfaces of the countertops and of the dental unit switches in ten Italian dental clinics with 640 and 630 CFU/100 cm^2^ respectively [7]. The bacterial contamination in the air of waiting rooms of this study ranged from 118 to 732 CFU/m^3^. Similar results were found in the air of dental consulting rooms with a wider range when the number of samples increases: from 180 to 490 CFU/m^3^ in two Poland dental offices [3] and from 2 to 2614 CFU/m^3^ in ten Italian dental offices [7]. The fungal contamination in the air of waiting rooms of this study ranged from 32 to 806 CFU/m^3^. Slightly lower contamination was found in the air of dental consulting rooms: from 10 to 340 CFU/m^3^ sampled nearly 25 dental units [11]. The contamination is significantly associated with the clinical activity [7].

The role that environmental contamination plays in the transmission of healthcare-associated infection is poorly understood. The cleaning and disinfection of healthcare environments and medical devices associated with hand hygiene are a higher priority of infection control [5]. In this study, the offices with daily deep wet cleaning had surfaces significantly less contaminated by fungi than others. A daily detergent-based cleaning of surfaces enables a reduction of both microbial amount and growth [12]. This domestic cleaning plays a role in the control of healthcare-associated infections [13]. Moreover, these actions of cleaning and disinfection are crucial issues that require attention to prevent the spread of antibiotic-resistant bacteria in healthcare facilities [14].

The qualitative analysis of this study presents similar results to the microbiological composition of aerosols sampled in other dental offices [1]. *Staphylococcus* and *Micrococcus* genera are mainly found in the air of dental offices, and *Pseudomonas* spp. were also frequently identified [3,7,10,15,16]. Regarding fungal contamination, *Penicillium* spp., *Cladosporium* spp., *Alternaria* spp., and *Aspergillus* spp. were the most quantified fungi in the air of dental offices [11,17]. The same micro-organisms were predominantly identified in hospitals [18,19].

In springer, *Bacillus* spp., *Staphylococcus* spp. and *Micrococcus* spp.—three bacterial genera identified in this study—are largely present both in outdoor and indoor air [20]. *Cladosporium* spp. and *Penicillium* spp.—the main quantified fungi in this study—were the most frequently identified fungal genera both in indoor and outdoor air of healthcare facilities [17,19]. A lot of human commensal bacteria of the skin such as *Micrococcus* spp., *S. epidermidis* and *S. hominis* were identified in the air and on the surfaces sampled. In some dental consulting rooms, bacteria such as *Pseudomonas aeruginosa* and *Stenotrophomonas maltophilia* were identified: these bacteria may originate from the water of the dental units [21,22]. In this study, no oral bacteria originated from the dental plaque were identified, but they may be found in dental consulting rooms [10]. Therefore, the micro-organisms indoor originated from two main sources: the outdoor air and the anthropogenic sources such as people from their skin, their clothing, and their respiratory systems [23,24]. They can also originate from healthcare activities or from other environmental sources such as water, notably in dental consulting rooms which are largely contaminated by aerosols or spatters containing micro-organisms originate from the dental unit [22].

Regarding antibiotic resistance, only a few antibiotic-resistant bacteria were detected in GP offices in this study: *S. aureus* resistant to penicillin, *S. aureus* resistant to penicillin and erythromycin, and *S. haemolyticus* resistant to methicillin and erythromycin. No antibiotic-resistant bacteria were found in dental offices. Four previous studies had identified antibiotic-resistant bacteria in dental offices: *S. maltophilia* resistant to trimethoprim were found in an air sample of a dental waiting room [10], and *S. aureus* resistant to methicillin were found on the surfaces of dental consulting rooms [4,8,9]. Several *Staphylococcus* species resistant to antibiotics (mainly resistant to penicillin, gentamicin and erythromycin) were isolated from the oral cavity: mainly *S. aureus*, but also numerous coagulase-negative staphylococci including *S. haemolyticus* [25]. Therefore, the oral cavity should be considered to be a potential reservoir and source of antibiotic-resistant bacteria spread in the environment. Methicillin-resistant *Staphylococcus aureus* present in the indoor environment may also originate from nasal colonization of healthcare workers [8,9]. 

The antibiotic resistance is a growing concern which spread not only in hospital but in independent healthcare facilities, too. In France, antibiotics are mainly prescribed for outpatients: GPs are responsible for 70% of all antibiotic prescriptions and dentists for 12% [26]. To tackle antibiotic resistance, several actions must be improved in dental and GP offices. First, the prescriptions should be improved to reduce the misuse and the overuse of antibiotics in dental and GP offices [26,27,28]. To achieve this, several measures have been already put in place in France through antibiotic stewardship programs [29]. For example, the elaboration of good practice recommendations for GPs [30] and for dentists [31], the setting up of regional antibiotic stewardship network (e.g., *AntibioEst*) [32], the implementation of a computerized decision support system for antibiotic prescription in primary care (e.g., *Antibioclic*) [33], and the creation of a website (e.g., *Antibio’Malin*) and of general public campaigns to increase awareness and provide information on antibiotics and antibiotic resistance among patients and healthcare workers [34,35]. Secondly, infection prevention and control measures are crucial to reduce the use of antibiotics. On the one hand, it includes infection preventive measures such as the vaccination [36] and, in dental offices, the promotion of the oral hygiene to reduce tooth decays and oral infections [37]. On the other hand, it includes measures to control the transmission of antibiotic-resistant bacteria. Knowledge and compliance with recommended infection-prevention procedures including suitable cleaning measures of environment (such as the doctor’s desk and examination table) and reusable medical equipment (such as stethoscopes) allow the reduction of the prevalence of antibiotic-resistant bacteria [14]. The contamination of surfaces by antibiotic-resistant bacteria in healthcare facilities such as dental offices may cause healthcare-associated infections [4]. The management of the environment and equipment including cleaning and disinfection activities is a crucial issue that requires attention to prevent the spread of antibiotic-resistant bacteria in healthcare facilities [12,38]. Moreover, healthcare worker hands may be colonized by antibiotic-resistant bacteria originate from the contaminated surfaces [39]. The improvement of hand hygiene compliance allows a decrease in the dissemination of antibiotic-resistant bacteria in the environment and to reduce the number of healthcare-associated infections [40]. In dental and GP offices, the infection prevention and control measures need to be improved because, to date, awareness and knowledge associated with measures to control the transmission of antibiotic-resistant bacteria among French healthcare workers are insufficient [41].

A limitation of this study was the microbiological diversity of healthcare offices was not totally explored. In the one hand, the culture medium and growth conditions used did not allow the identification of all types of organisms, including viruses, anaerobic bacteria, and organisms requiring specialized medium [15]; the culture-based method used was limited to the research of culturable micro-organisms [24]. In the other hand, 30% of bacteria genera and 2% of fungal genera were not identified, partially due to the limits of identification methods [42]. However, this study presents several strengths: this is the first—to the author’s knowledge—which study environmental contamination in GP offices. A great number of samples were performed in each facility both on surfaces and in the indoor air, it provides a wide qualitative and quantitative description of bacterial and fungal contamination in dental and GP offices. In addition, antibiotic resistance of bacterial pathogens was studied.

Since the COVID-19 pandemic, it was recalled that all surfaces in healthcare facilities should be regularly cleaned and disinfected (especially high-touch surfaces) and that an adequate ventilation with fresh outdoor air was required to reduce the indoor air contamination [43]. This study was performed before the COVID-19 pandemic. The microbiological contamination on the surfaces and in the indoor air of healthcare facilities have possibly evolved since the COVID-19 recommendations. The initial encouraging results of this study need further investigation, including more healthcare facilities.

## 4. Materials and Methods

### 4.1. Settings

This study was conducted in five dental offices and five GP offices located in Eastern France. These offices were recruited on a voluntary basis. Samples and measurements were taken in May and June 2019. 

Deep wet cleaning (including the washing and the scrubbing of the floors with a detergent product) was carried out once a day—before or after the occupancy period—in all dental offices (5/5). It was carried out daily (1/5), biweekly (3/5), or weekly (1/5) in GP offices. Concerning ventilation, 40% of offices had an air-conditioning system, and 40% had mechanical ventilation with a mean air exchange rate of 0.4 ± 0.2 volumes per hour. 

### 4.2. Sampling Strategies

For each healthcare facility, samples were collected during a typical day of care activity. The areas sampled were waiting, consulting, and sterilization rooms. No patient was present in the sampled rooms.

Six surface samples were collected in each room: four flat surfaces and two non-flat surfaces. Flat surfaces were sampled by agar contact plates (25 cm^2^) through an applicator with uniform pressure of 600 g for 10 ± 0.5 s (BIOcontact^®^ L6, AC-SPerhi, Saint-Laurent-des-Arbres, France). Non-flat surfaces were sampled in each room using swabs with tubes containing 10 mL of COPAN SRK^®^ solution (COPAN, Murrieta, CA, USA), which contains a disinfectant inhibitor. The samples were taken from surfaces generally not disinfected between each patient to collect micro-organisms present in these healthcare environments. Flat surfaces were chair seat, chair back, table, and magazine cover in waiting room; cabinet door, work surface, desk, and examining table or arm of the X-ray generator in consulting room; cabinet door, work surface, autoclave door, and washer-disinfector door in dental sterilization room. Non-flat surfaces were toys and door handle in waiting room; diaphragm of the stethoscope and inside the blood pressure cuff in the GP consulting room; space bar on the keyboard and arm of the operating lamp in dental consulting room; valve handle and light switch in dental sterilization room.

One indoor air sample (0.5 m^3^) was collected in the waiting room using single-stage MAS-100 NT^®^ impactor (Merck KGaA, Darmstadt, Germany) at a flow rate of 100 l/min for five minutes. The impactor was disinfected before each sampling, and it was positioned more than one meter from the floor and walls.

### 4.3. Microbiological Analysis

Bacteria were cultured on trypticase soy agar (TSA) contact plates and on plate count agar (PCA) for swabs and indoor air samples. Fungi were cultured on Sabouraud chloramphenicol agar (SAB). All petri dishes were incubated for five days at 30 ± 2 °C and 25 ± 2 °C for bacteria and fungi, respectively. Colony growth was checked daily. Concentrations were expressed as colony-forming units (CFU) per 100 cm^2^ for contact plates and CFU/m^3^—using positive-hole corrections—for air samples.

Bacteria were identified using Gram staining and biochemical analytical profile index (API) tests (bioMérieux, Marcy-l’Etoile, France). Antibiograms were performed in accordance with the guideline of the European and French committees on antimicrobial susceptibility testing [44] to test the antibiotic susceptibility of bacterial pathogens. Fungi were identified according to their macroscopic and microscopic morphology stained with lactophenol.

In each facility, field blank samples were collected, then incubated and analyzed to assess whether the samples may have been contaminated. No micro-organism was detected in the field blank samples.

### 4.4. Statistical Analysis

Data were described as numbers and percentages for categorical variables and as means ± standard deviations or median associated with range for continuous variables. Due to non-normal distributions of the data (analyzed using the Shapiro–Wilk test), all the results were statistically processed with the Kruskal–Wallis and Mann–Whitney tests using RStudio^®^ (RStudio Inc., Boston, MA, USA) version 1.1.456. Statistical significance was fixed at *p* < 0.05.

## 5. Conclusions

The microbiological contamination in dental and GP offices seems low. The identified bacteria and fungi seem mainly originate from the outdoor air and from the humans present inside. A few pathogenic micro-organisms and rare antibiotic-resistant bacteria have been identified. These micro-organisms present a risk of healthcare-associated infection. The cleaning and disinfection activities in healthcare environments are crucial to reducing the microbiological risk. The identification of a few antibiotic-resistant bacteria highlights the need for continued surveillance and infection control practices associated with judicious antibiotic use to tackle this growing problem.

## Figures and Tables

**Table 1:** Micro-organisms isolated from air and surface samples of dental (D) and general practitioner (GP) offices.

Micro-Organism		Air		Surfaces					
		Waiting Rooms		Waiting Rooms		Consulting Rooms		Sterilization Rooms	
		D	GP	D	GP	D	GP	D	GP
**Gram-positive cocci**									
	*Kocuria kristinae*	-	-	-	-	-	-	+	NA
	*Kocuria* spp.	-	-	+++	++	-	++	+	NA
	*Kocuria varians/rosea*	+	-	++++	++++	-	+++	+	NA
	*Micrococcus* spp.	++++	+++++	+++++	+++++	+++++	+++++	++++	NA
	*Staphylococcus aureus*	-	+ ^R^	-	+ ^R^	-	-	-	NA
	*Staphylococcus capitis*	-	-	-	-	-	+	-	NA
	*Staphylococcus chromogenes*	-	-	+	-	-	+	+	NA
	*Staphylococcus cohnii*	++	+	+	-	-	+	-	NA
	*Staphylococcus epidermidis*	-	-	++	+	++	++	+++	NA
	*Staphylococcus haemolyticus*	-	+ ^R^	-	+	-	+	-	NA
	*Staphylococcus hominis*	-	-	+	+	-	+	-	NA
	*Staphylococcus sciuri*	-	-	-	+	-	+	-	NA
	*Staphylococcus* spp.	+++	+++	++++	++++	++++	+++	+++	NA
**Gram-negative bacilli**									
	*Brevibacillus* spp.	-	-	-	-	-	+	+	NA
	*Mannheimia haemolytica*	-	-	+	-	-	-	-	NA
	*Moraxella* spp.	-	-	-	-	-	+	-	NA
	*Pantoea* spp.	-	-	-	+	-	-	-	NA
	*Pasteurella* spp.	-	-	-	-	-	+	-	NA
	*Proteus penneri*	-	-	-	-	-	-	+	NA
	*Pseudomonas aeruginosa*	-	-	-	-	+	-	-	NA
	*Pseudomonas luteola*		+	-	-	-	+	-	NA
	*Psychrobacter phenylpyruvicus*	-	-	-	-	+	-	-	NA
	*Stenotrophomonas maltophilia*	-	-	++	+	++	-	+	NA
	*Sphingomonas paucimobilis*	-	-	+	-	-	-	-	NA
**Endospore-forming Gram-positive bacilli**									
	*Bacillus cereus*	-	-	-	-	-	-	+	NA
	*Bacillus smithii*	-	-	-	-	+	-	-	NA
	*Bacillus* spp.	-	+	+	++	-	++	-	NA
**Filamentous fungi**									
	*Acremonium* spp.	-	-	-	-	-	+	-	NA
	*Alternaria* spp.	++	+	++++	+++	++++	+++++	+++	NA
	*Aspergillus flavus*	-	-	-	+	-	-	-	NA
	*Aspergillus fumigatus*	+	-	+	-	-	-	-	NA
	*Aspergillus niger*	+	-	-	+	+	-	-	NA
	*Aspergillus ochraceus*	++	++	+	-	+	-	+	NA
	*Aspergillus* spp.	+	-	+	-	++	-	-	NA
	*Aspergillus versicolor*	+	-	+	-	-	+	+	NA
	*Aureobasidium* spp.	-	-	+	-	-	+	-	NA
	*Chaetomium* spp.	-	-	-	-	+	-	-	NA
	*Cladosporium* spp.	+++++	+++++	+++++	+++++	+++++	+++++	+++++	NA
	*Eurotium herbarorium*	-	+	+	+++	-	-	-	NA
	*Mucor* spp.	-	+	+	++	+	-	-	NA
	*Penicillium* spp.	+++++	+++++	++++	+++++	+++++	+++++	+++	NA
	Phylum Basidiomycota	+++	+++++	+++++	++++	++++	+++++	++++	NA
	*Rhizomucor* spp.	-	-	-	-	+	-	-	NA
	*Rhizopus* spp.	-	-	+	+	-	-	-	NA
	*Trichoderma* spp.	-	-	-	-	+	-	-	NA
	*Ulocladium* spp.	-	-	-	+	-	-	-	NA
**Yeasts**									
	*Rhodotorula* spp.	+	+++	++	++	-	-	++	NA
	Other yeasts	+++	+	++++	+++++	++++	++++	++	NA

Notes: “+” to “+++++” indicate the presence of the identified specie or genus in 1 to 5 offices; “-” indicate the absence of the specie or genus in all sampled facility rooms; “^R^” indicate the antibiotic resistance of the identified bacterial specie; NA, not applicable.

## Data Availability

The datasets supporting the conclusions of this article are included within the article and its additional file.

## Supplementary Materials

The following are available online at https://www.mdpi.com/article/10.3390/antibiotics10111375/s1, Table S1: Comparison of the median microbiological contamination of surface (CFU/100 cm^2^) and air samples (CFU/m^3^) of dental (D) and general practitioner (GP) rooms, Table S2: Comparison (*p*-value) of the median microbiological contamination of surfaces between the rooms of dental and general practitioner (GP) offices.
