# Supplementary material for: Microbiological Contamination of the Office Environment in Dental and Medical Practice
Source: Antibiotics (Basel). 2021 Nov 10;10(11):1375. doi: 10.3390/antibiotics10111375 (PMC8614722; doi:10.3390/antibiotics10111375)
Supplement: Supplementary file 1 [file antibiotics-10-01375-s001.zip › antibiotics-1447406-supplementary.pdf]

antibiotics

MDPI

Supplementary Materials

Microbiological Contamination of the Office Environment in Dental and Medical Practice

Table S1. Comparison of the median microbiological contamination of surface (CFU/100 cm^2) and air samples (CFU/m^3) of dental (D) and general practitioner (GP) rooms.

Contamination	Air			Surfaces								
	Waiting rooms			Waiting rooms			Consulting rooms			Sterilization rooms		
	D	GP	*p*-value	D	GP	*p*-value	D	GP	*p*-value	D	GP	*p*-value
Bacteria	382	424	1	114	148	0.7	102	140	0.2	118	NA	NA
Fungi	112	460	0.3	28	64	0.3	14	44	0.07	8	NA	NA

Table S2. Comparison (*p*-value) of the median microbiological contamination of surfaces between the rooms of dental and general practitioner (GP) offices.

Contamination	GP's offices	Dental offices		
	Waiting *vs* consulting rooms	Waiting *vs* consulting rooms	Waiting *vs* sterilization rooms	Consulting *vs* sterilization rooms
Bacteria	0.9	0.5	0.8	0.8
Fungi	0.5	0.1	0.04	0.7
